# Development of Resistance Spot Welding Processes of Metal–Plastic Composites

**DOI:** 10.3390/ma14123233

**Published:** 2021-06-11

**Authors:** Paweł Kustroń, Marcin Korzeniowski, Tomasz Piwowarczyk, Paweł Sokołowski

**Affiliations:** Department of Metal Forming, Welding and Metrology, Faculty of Mechanical Engineering, Wroclaw University of Science and Technology, Wyb. Wyspianskiego 27, 50-370 Wroclaw, Poland; pawel.kustron@pwr.edu.pl (P.K.); marcin.korzeniowski@pwr.edu.pl (M.K.); tomasz.piwowarczyk@pwr.edu.pl (T.P.)

**Keywords:** metal–plastic composites, Litecor, resistance spot welding, joining, ultrasound, shunt current, induction heating

## Abstract

Metal–plastic composites (MPCs) are gaining importance mainly due to high strength to weight ratio. They consist of three layers, two outer metallic cover sheets, and a plastic core. The presence of that inner plastic layer makes them rather unsuitable for joining by means of any conventional welding processes, which significantly reduces the application range of MPC. In this work, three various resistance spot welding (RSW)-based concepts were developed to overcome that limitation and join Litecor to DP600 steel. In all cases, a dedicated initial stage was implemented to RSW, which was aimed at removing the non-conductive polymer layer from the welding zone and creating the proper electrical contact for the resistance welding. These were, namely: (i) shunt current-assisted RSW; (ii) induction heating-assisted RSW; and (iii) ultrasonic-assisted RSW. The development of each concept was supported by finite element modeling, which was focused on setting the proper process parameters for polymer layer removal. Finally, the macro- and microstructure of exemplary RSW joints are shown and the most common spot weld features as well as the further development possibilities are discussed.

## 1. Introduction

Metal–plastic composites (MPCs) have many advantages that are particularly desired by manufacturers who want to produce lightweight structures. The most important include low weight compared to steel while maintaining high strength parameters, the possibility of plastic forming using standard cold forming processes, and lower costs compared to competitive aluminum alloys. This is one of the reasons why the automotive and aviation industries are interested in this group of materials. The problem that prevented the immediate implementation of MPC materials was the lack of appropriate technologies for joining these materials with each other as well as with other materials (steel, aluminum). Conventional thermal joining techniques (arc and laser welding, resistance welding) were not applicable due to the presence of a polymer layer. On the other hand, the proposed alternative technologies (clinching, blind riveting) did not ensure the appropriate quality of joints. The challenge is to develop a joining technology that allows for the effective joining of MPCs with thermal techniques. Resistance spot welding of MPC materials is relatively new joining technology, which still needs to be developed and optimized.

The joining of polymer composite materials to metal alloys is quite demanding, and is currently mainly carried out by mechanical fastening (e.g., self-piercing riveting, clinching), joining by forming (e.g., hemming, seaming), and adhesive bonding [1,2,3,4,5]. However, these methods show some disadvantages, particularly important in an industrial production process: introducing stress concentrations, delamination during machining (mechanical fastening), no lap joints, complex and costly industrialization (joining by forming) or extensive surface preparation requirements, long curing time, and surface tension issues (adhesive technology) [2,3,6]. Due to the high application potential and numerous advantages of resistance welding, this joining method can be applied for joining various types of composite elements. For example, Naik et al. [7] have shown that manufacturing of high strength metal–polymer–metal sandwich panels is possible using resistance spot welding for joining wire mesh at the interface between the metal sheet and polymer core. However, the presence of the polymer intermediate layer in metal–plastic-composites (MPCs) considered in this publication causes a technological challenge for resistance welding, especially direct resistance spot welding (RSW).

Nevertheless, composite manufacturers claim that this is possible [4,8,9,10], which has been later confirmed by scientific research. In some studies [1,3,11], it is emphasized that resistance spot welding of MPC laminates is not possible without modifying the joint, and on the advice of the manufacturer, they suggest using a third electrode (shunt) to melt away the polymer core to achieve electrical contact between two metal layers. Therefore, one of the most detailed studies on this topic has been carried out by Naimi et al. [12]. In this work, the idea of using a shunting element for resistance spot welding of Litecor with DP800 cover sheets and resolving the non-conductivity problem of a polymer core was also used. The authors describe both the optimal technological parameters (electrode force, welding current, welding time, number of pulses, distance between the electrode and the shunt tool) as well as the results of tests carried out on the joints (fracture load in tensile shearing test, hardness in the fusion zone and HAZ, macro/micrograph and SEM/EDS examinations). Shunt current-assisted resistance spot welding was also analyzed by Tanco et al. [13], where the authors developed a technology for resistance spot welding three sheets of DC06 1.5 mm + Litecor 0.8 mm + DC06 1.5 mm by selecting process parameters that allowed for good quality joints to be obtained. Numerical simulations were performed in SORPAS^®^ 3D and the compatibility of the diameter of the spot welds were compared to the experimental results. Results presented in both articles [12,13] indicate the high industrial potential of the proposed method of joining. However, obtaining the required quality of joints, the welding condition, and parameters must be precisely controlled.

There have also been attempts at the resistance welding of nonmetallic (i.e., non-conductive) composite materials with metal alloys. Many scientific works have shown that this is still possible by using an additional heating element (which has a higher resistance), usually a stainless steel mesh, placed between the sheet interface. This method is also known as resistive implant welding, electrical-resistance fusion, or electro-fusion [14]. In [15], an investigation of the resistance welding between thermoplastic matrix composites of carbon fiber reinforced polyetherimide and aluminum substrates (7075-T6 grade alloy) was presented. It has been shown that the resistance welding of 7075-T6 aluminum alloy/carbon fibre reinforced polyetherimide (CF-PEI) joints can lead to consistent lap shear strength values greater than 20 MPa (laminate cohesive failure). On the other hand, the large amount of heating energy provided to the specimen caused significant fiber motion and deconsolidation in the CF-PEI arm of the specimen, and turned out to be a problem during the resistance welding of these materials. Similarly, in [16], carbon fiber/polyetheretherketone (PEEK) composites and 7075-T6 aluminum were resistance welded. The problem of contact of the heating element with aluminum was eliminated by using an additional polymer layer between the welding surfaces. An extensive surface treatment of the aluminum substrate was also suggested prior to bonding to increase the joint quality. In other studies [17], the adhesion between a thermoplastic matrix (i.e., polyphenylene sulfide) and stainless steel mesh heating element was evaluated and improved through the application of surface coatings on stainless steel. A more efficient heating element/PPS adhesion was achieved here through the development of a silane coating applied to the stainless steel mesh heating element (with an improvement of 32% when compared to the joints welded by using an untreated heating element). The authors of the publication [18] went a step further and suggested a new heating element made of polyetherimide, rendered electrically conductive by the addition of 10% wt. multiwall carbon nanotubes.

Taking into account the limitations during the thermal joining of MPCs, some novel approaches are currently proposed in the literature (e.g., with mechanical nuggets aimed to produce lap joints in metal-polymer sandwich composite sheets) [19]. The process involves drilling a blind hole and removing the upper metal cover sheet and the polymer core. Then, the MPC sheets may be joined together by compressing a metal insert placed in-between. The limitations of conventional welding techniques like arc welding, laser welding, and resistance spot welding mean that the feasibility of friction stir welding to join thin sandwich components has also been studied [20]. Another idea was proposed in [21], in which hybrid joints obtained by combining adhesion and clinching were investigated. Finally, recent findings in [22] led to the conclusion that the polymer layer in such MPC materials can be displaced, or even removed, in many different ways. However, the main focus was on ultrasonic-assisted displacement of the polymer layer and it was proven that this can be a proper direction for the further development of various joining processes of MPCs.

As presented above, the resistance welding of non-conductive to conductive materials is already possible, however, some modifications to conventional processing are required. In this work, the development of RSW-based joining of metal–plastic composites to steel is presented. The limitation in joining MPCs to other materials was omitted here by introducing three different approaches: shunt current-assisted RSW, induction heating-assisted RSW and ultrasonic-assisted RSW.

## 2. Experimental Methodology

### 2.1. Materials

The RSW-based processes were developed for the joining of Litecor^®^ to DP600 steel. Litecor, a sandwich material manufactured by ThyssenKrupp Steel Europe, Duisburg, Germany, was used for the tests. Litecor is a compromise between steel and aluminum in terms of weight and economy [3,8,23].

**Figure 1:**
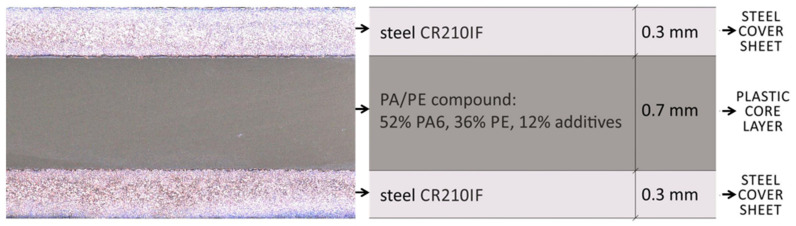
Overview of the MPC material Litecor^®^ used in this research.

Litecor characterized by a tensile strength in the range of 190–240 MPa (confirmed by own research, *R_m_* = 230 MPa) and a yield point in the range of 120–180 MPa with an elongation A_80_ of about 28% [11,24]. It consists of three layers (for macrostructure and schematic representation see Figure 1) created in the warm roll bonding process [3]. The 0.2–0.5 mm thick outer claddings are made of HX220YD steel according to EN 10346 (HC220Y according to EN 10268, CR210IF according to VDA 239-100), which is a high-strength IF (interstitial-free) cold rolled steel suitable for forming [3,4,9,11,23,24,25,26,27].

The chemical composition of HX220YD steel is summarized in Table 1, and its basic mechanical properties in Table 2 [25,27,28]. The steel is additionally protected against corrosion (zinc layer, ZE75 or ZE50) [4,24]. While the inner layer is a polyamide/polyethylene compound (0.3–1 mm), it consists of 52 wt.% PA6, 36 wt.% PE, and 12 wt.% other additives [3,4,9,11,23,24]. Properties of PE/PA-compounds important in the aspect of the future connection are mainly: melting temperature of 220 °C, solidification/crystallization temperature of 192 °C, and decomposition temperature above 300 °C [24]. Litecor comes in two variants: Litecor C (classical, used in this research) and Litecor S (strong) [11,24].

Sandwich material Litecor was joined with 0.8 mm thick sheet steel DP600. It is a two-phase steel containing soft ferrite for good formability and hard martensite for high material strength. DP600 is a cold-rolled steel with a formability that makes it suitable for deep drawn components. This steel has good weldability and is suitable for car safety components. The chemical composition of DP600 steel is presented in Table 1, and its basic mechanical properties in Table 2.

### 2.2. Welding Approach and Equipment

**Figure 2:**
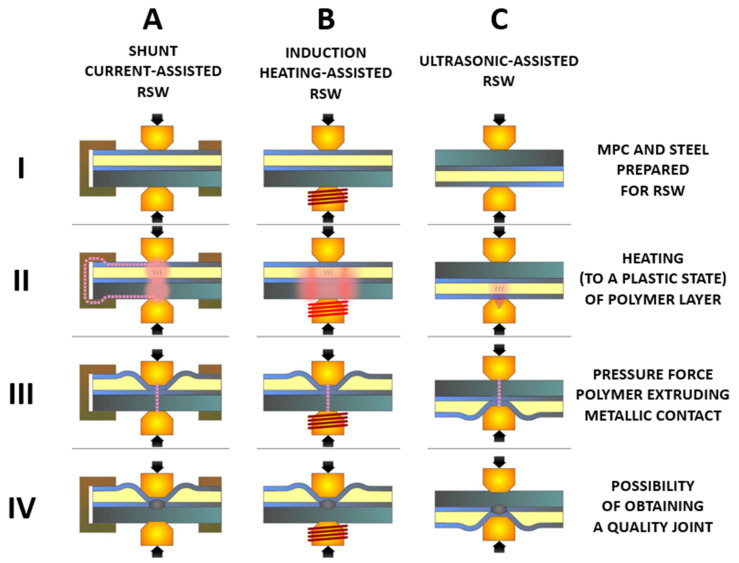
The schematic representation of the three concepts of RSW-assisted technology for MPC joining: (**A**)—shunt current-assisted RSW; (**B**)—induction heating-assisted RSW; and (**C**)—ultrasonic-assisted RSW. Each process involves four stages (I to IV, described in the figure).

The biggest limitation during resistance spot welding of MPC composites is the presence of a non-conductive core, which limits (or even prevents) the flow of electric current through the material. To ensure the current flow through the external metal sheets, the non-conductive polymer must be removed in the first stage of the process. As part of the work, three concepts of polymer removal were tested: (i) electric current shunt circuit; (ii) induction heating; and (iii) high intensity ultrasonic waves. These methods were aimed at removing the non-conductive polymer layer from the welding zone and creating the proper electrical contact for the resistance welding process. The ideas behind these three processing routes are as follows:

- Shunt current-assisted RSW (Figure 2A)—in the first stage of the process, the electrical current flows through the circuit with lower resistance (shunt element made of Al alloy) and bypasses the polymer layer. Due to the Joule–Lenz Law, the work of the flowing electric current is converted into heat. The heat generated in the shunting element is transferred into the external cladding of the MPC material, which in turn causes the polymer core to be heated. Thus, under the influence of the electrodes, the pressure is squeezed out of the cladding. After the metal cladding makes contact with each other, the process of weld formation occurs. The current path is significantly shortened and the electric current flows through the metallic contact surface. The highest resistance is in the contact interface between the electrode surface and metal clad/steel sheets. For that reason, these areas are rapidly heated. The liquid welding nugget and consequently a permanent joint may be formulated.

A 3 mm thick Al alloy flat bar bent at an angle of 180° was used as a shunt circuit tool. To ensure the current flow through it, a shunting element was initially pressed into the material to be welded.

- Induction heating-assisted RSW (Figure 2B)—the bottom electrode of the spot welding gun is overlapped by the induction coil, which generates a magnetic field with high intensity. The magnetic field generated by the inductor in the proximity of conductive materials generates eddy currents inside them. The spot welding process occurs in two stages. The inductor is switched on first. The magnetic field generated by the inductor causes the eddy currents to flow through the bottom electrode and the DP600 steel. Then, the heat generated in DP600 steel is transferred into the MPC, which causes its heating. The heating ratio depends on the power of the inductor. After the required time (determined empirically during the heating tests), the inductor is turned off and the resistance welding stage occurs. The heat generated by the eddy currents and transferred into the MPC plasticizes the polymer core, which, under the influence of the electrode pressure, gets squeezed from the cladding. Thus, the electric current is able to flow through the contact surface and the further process of spot joint formulation occurs. For induction heating, a high power (40 kW, 22.5 kHz) inductor has been used. The coil was made of high conductive copper pipe, with 3.5 mm internal diameter, 5mm external diameter, and three turns. Thee internal coil diameter was 45 mm.
- Ultrasonic-assisted RSW (Figure 2C)—this process consists of combining two physically different ways of heating materials during one welding cycle, in two successive stages. In the first phase of the process, the polymer core is heated by the active interaction of high intensity ultrasonic waves and the polymer is extruded by the applied pressure force by a resistance spot welding gun. In the second phase, the welding current is switched on. It flows through the clean metallic interface, which causes resistive heating of the steel cladding and eventually a permanent connection of MPC and steel is obtained.

In this approach, the welding circuit has been extended to the high power sandwich type ultrasonic transducer. The force application is performed by a resistance spot welding gun (i.e., pressing the upper electrode moving along the vertical axis). However, it needs to be underlined that the lower electrode is supplied with a high power ultrasonic system combined with an electric circuit. It consists of an ultrasonic transducer with a resonance frequency of 20 kHz and a maximum power of 3 kW, a booster, which is an intermediate element between the converter and the working tool. The booster’s geometry is designed in such a way that its rigid mounting on the current rail does not attenuate the ultrasonic vibrations of the working tool, the so-called sonotrode. The sonotrode is mechanically coupled to the booster and it is a key element of the developed welding setup under the third approach.

In each of these three cases, a stationary inverter 10 kHz pedestal resistance spot welding gun with the active power of 40 kVA and the range of pressure force between 0.8–4.5 kN was used.

### 2.3. Numerical Modeling Methodology

To better understand the physical background of the discussed welding processes, the development of each RSW concept was supported by means of numerical modeling. The studies conducted by finite element modeling (FEM) allowed us, first of all, to select the appropriate strategy for the preheating and the preliminary selection of the resistance welding parameters. As a part of this research, all three preheating techniques were analyzed by the FEM method.

The first discussed method was using a shunt element that allowed for a relatively low current to be flown between the welding electrodes. This resulted in the generation of heat at the contact surfaces, especially at the electrode–material interface. Due to the generated thermal energy, the polymer core heats up and reaches a viscoelastic state, thanks to which it can be formed and removed from the welding zone. This process continues until the metal cover sheets of the MPC come into contact. This is where the commonly used resistance spot welding process can be further carried out.

The main problem during a polymer layer heating is its very low thermal conductivity. As a result, the time needed to raise its temperature is relatively long compared to the time of the resistance spot welding process. Attempting to perform this process too quickly typically causes the coating material to overheat, burn the polymer, or even expulsion due to the appearance of large amounts of gases (polymer burn products).

**Figure 3:**
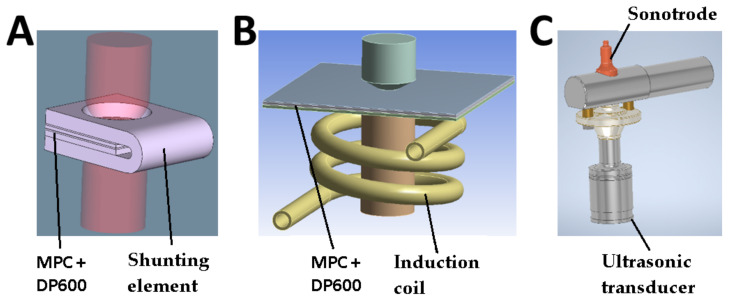
The overview of the model geometries: (**A**) shunt current-assisted RSW; (**B**) induction heating-assisted RSW; and (**C**) ultrasonic-assisted RSW.

In this case, numerical simulation was used for explaining the physical background of this process and setting the correct heating time by studying the temperature distribution inside the weld area. With this information, the process can be quickly optimized. The FEM calculations were made using Simufact Welding software, dedicated to the simulation of various types of welding processes (e.g., resistance spot welding). The most important initial and boundary conditions used in the FEM model (Figure 3A) are as follows:

- Welding electrodes are supplied with multiple electric current pulses with a duration of 150 ms, hold time 620 ms, and intensity of 3–6 kA;
- The electrode clamping force was set at 1 kN throughout the entire process;
- Exchange of thermal energy between the elements of the welding system was applied by entering data on the convection coefficient, emissivity, and thermal conductivity of the materials (Table 3);
- The contact area of the shunt element and its minimum cross-section were designed to be at least 20 times larger than the electrode-material contact area. It is important to concentrate the heat production in the right place (i.e., in the welding area).

The induction process has a similar background to the described above methodology using a shunt element. In particular, it should be emphasized that the preheating mechanism is also based on the transfer of thermal energy from inductively heated MPC cover sheets to the polymer core. Due to the low thermal conductivity of the polymer and the long time needed to heat up the MPC core by induction heating, the heated area is relatively large.

The FEM calculations were carried out with the use of the Maxwell 3D module of the ANSYS software. The geometry of the model is shown in Figure 3B. The most important boundary conditions are as follows:

- Exchange of thermal energy between the elements of the welding system is described in Table 3;
- Induction coil excitation with 468 A current with a frequency of 22.4 kHz; and
- The air surrounding the system with a computational domain of 100 × 100 × 100 mm^3^.

The analysis allowed to illustrate the heating process and select the appropriate joint configuration and parameters of the preheating process. Two cases were considered: (i) the MPC material was located on the side of the induction coil and (ii) the steel sheet was placed on the side of the coil. The main aim was to determine the technologically more advantageous configuration of the joint fabrication and, above all, to reduce the risk of MPC material damage, its overheating, deformation, or delamination during the joining process.

The third method was based on an ultrasonic system integrated with a spot welder (Figure 3C). This method is aimed at eliminating the disadvantages of the previously described methods (i.e., shortening the preheating time and reducing the degradation of MPC cover sheet materials as well as increasing the quality of electrical contact of the electrodes with the material and then, the joined materials themselves).

**Figure 4:**
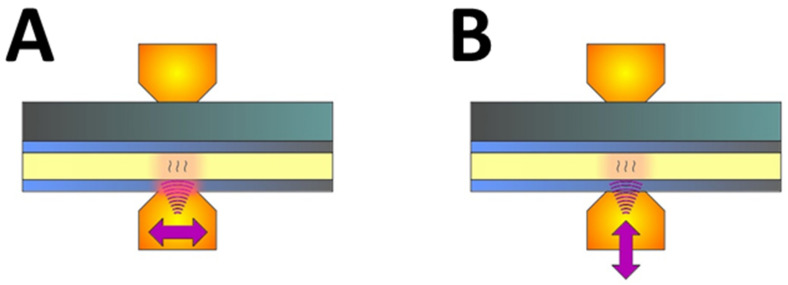
The two analyzed vibrating systems: (**A**) transverse, (**B**) longitudinal.

The FEM calculations were aimed at selecting the proper type of vibrations (longitudinal or transverse) that can be used in the polymer preheating process. In both cases, the viscoelastic heating phenomenon is considered, resulting from the internal friction of polymer molecules. Clarification of this issue required the comparison of both methods in terms of process ratio, efficiency, and temperature distribution of the joint during preheating. For this purpose, two vibrating systems were designed. The first one had a longitudinal direction (Figure 4A) and the second one had a transverse direction (Figure 4B) of vibration.

The model was made in the ANSYS coupled field transient environment, where a complex thermo-mechanical analysis was used, allowing for the use of a component called viscoelastic heating, as described by Equation (1):

(1) $$Q=f*\pi *\varepsilon_{0}^{2}*E^{\prime \prime }$$

where *Q* is the thermal energy [J]; *f* is the vibration frequency [Hz]; *ε*_0_ is the amplitude of the strain; and *E*″ is the loss modulus.

It is especially important to properly implement the Young’s modulus composed of a loss modulus and a storage modulus (Equation (2)) into the Prony material model, according to [29,30]. Thanks to this, it is possible to determine the amount of thermal energy generated in the polymer core:

(2) $$E=\left(\frac{\sigma_{0}}{\varepsilon_{0}}\right)\left(\sin\left(\delta \right)+i\cos\left(\delta \right)\right)=E^{\prime }+iE^{\prime \prime }$$

where *E*′ is the storage modulus and *E*″ is the loss modulus; *σ*_0_ is the amplitude of the stress; *ε*_0_ is the amplitude of the strain; and *δ* is the lag angle of the stress and strain.

A time-dependent sinusoidal displacement was used as the load. The frequency of forced vibration was 20 kHz and its amplitude was equal 50 μm. In addition, a pressure of 10 kPa was applied as a representation of the clamping force of the electrode and the sonotrode. The lower electrode is represented by the fixing, where the degrees of freedom were blocked in the form of translation in the x and y directions. The most important boundary conditions and material properties used in the models are described in Table 3.

### 2.4. Metallographic Examination

Finally, the macro- and microstructures of the manufactured joints were observed. This was done as a first assessment of fabricated spot welds to see the effect of joining concept and the parameters tested on the spot weld formulation mechanism, polymer core removal from the welding zone, and the occurrence of welding defects. The main focus was paid on the weld nugget morphology, the heat affected zone size, and overall quality of the weld. The surfaces of as-welded samples, side edges, and metallographically prepared cross-sections (i.e., ground, polished, and etched with 2% Nital) were observed by light microscopy (Keyence VHX-6000, Keyence, Osaka, Japan).

## 3. Results

The development of various RSW-based joining concepts of metal–plastic composites are discussed in the following three subsections.

### 3.1. Shunt Current-Assisted Resistance Spot Welding

**Figure 5:**
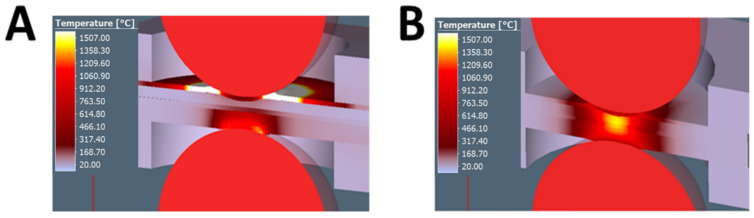
The results of the shunt current-assisted RSW FEM simulation: (**A**) preheating process and (**B**) welding process completed.

The results of the FEM numerical analysis of this welding process were aimed at developing an appropriate design of the current shunting element and the selection of the initial process parameters including heating and forming of the polymer MPC core. The conducted analyses showed that too rapid heating of the joined materials caused a strong degradation of the metallic cover sheets of the MPC. The thickness of one sheet was very low, equal to 0.3 mm. Hence, very fast overheating of this element occurred when compared to the thicker steel sheet DP600 (0.8 mm). Figure 5A shows a case where too fast heating was applied. In this case, the upper MPC outer layer was strongly overheated, deformed, and, as a result, the MPC material was delaminated. This was the effect of the relatively high resistance of this material, its low thermal conductivity, and the inability to quickly dissipate heat out of the welding area. In addition, the very high welding current applied in one pulse caused a strong degradation of the cover material, while the polymer layer did not reach the appropriate temperature to be removed from the welding zone. The correct welding process is presented in Figure 5B. After the proper formation (removal) of the polymer layer, the further occurrence of the welding process was similar to the well-known RSW.

**Figure 6:**
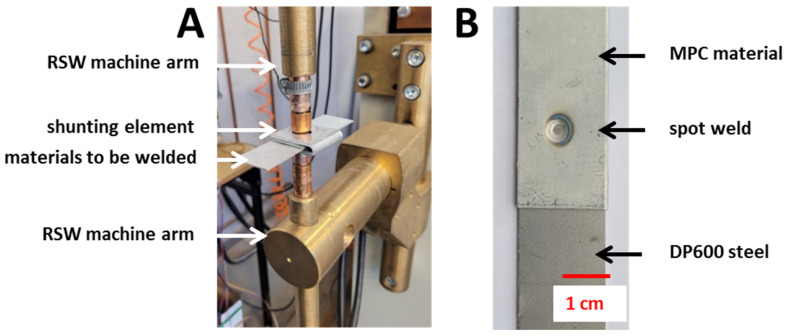
The presentation of: (**A**) sample with applied current shunting element during joining, (**B**) typical test sample, as welded.

The spot welds were manufactured by using the designed shunt element (Figure 6) and selected parameters obtained in the FEM simulations. During the research work, the FEM model was verified and the process parameters were finally selected. This made it possible to solve the problem of overheating the MPC material by using a multipulse welding program that consists of five to 10 preheating pulses with a small current value (approx. 3–4 kA). Then, after forming the polymer core, a final high-intensity current pulse (approx. 6 kA) was applied. This ensures a longer heating time, but a more uniform temperature distribution in the welding zone may also be obtained. In this way, it is possible to prevent the MPC material against thermal degradation and obtain a desired high-quality spot weld. The entire process of the weld formulation takes usually up to 5 s.

**Figure 7:**
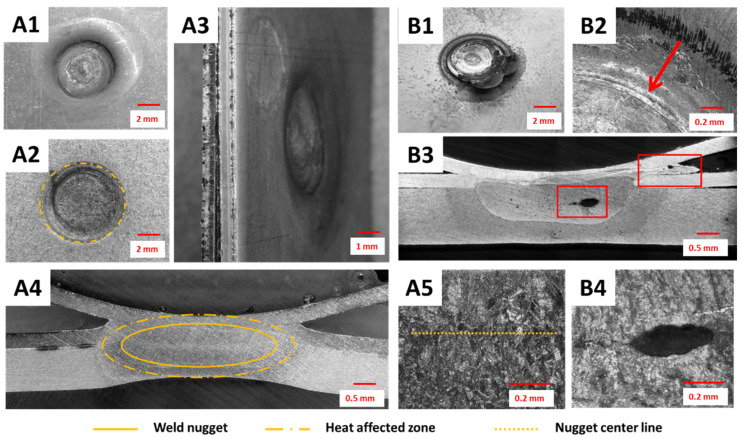
Examples of spot welds fabricated by shunt current-assisted RSW properly: (**A1**) top view, (**A2**) bottom view, (**A3**) side view, (**A4**) cross-section at low magnification, and (**A5**) weld nugget at high magnification) and non-properly formulated: (**B1**) top view, (**B2**) top view magnified, (**B3**) cross-section at low magnification, and (**B4**) weld nugget at high magnification.

The weld surfaces and shapes of nuggets obtained in the shunt current-assisted resistance spot welding are shown in Figure 7. The diameter of the obtained welds, measured after breaking the joint, was usually around 3.9 mm. By using this concept and selecting the proper process parameters, it was possible to form a defect-free spot weld (Figure 7A1–A5). The desired high quality of the joints was obtained without cracks in the outer metallic layer or visible polymer expulsions, as presented in Figure 7A1–A3. Then, the weld cross section revealed a properly established nugget, which included three layers of metal sheets and was not contaminated by a polymer core during the joint formulation (Figure 7A4,A5).

However, during the technology development, different non-conformities and defects were also observed. Both splash or cracking of the outer metal sheet in MPC, followed by an extensive polymer expulsion, were observed at the spot weld surface when the process parameters were not properly set (Figure 7B1,B2). This was usually observed when too long or too intensive heating of the shunt current was set, and in critical cases even caused overburning of the polymer layer. In such cases, further defects were observed at the RSW joint cross-section and then the nugget was not properly formed. Usually, the outer layer of the MPC cover sheet was fractured and the polymer flowed out or the middle steel layer was broken, and some expulsion of metal to the polymer occurred (Figure 7B3). Furthermore, some large shrinkage cavities were observed in the center of the weld nugget, which were followed by some randomly distributed micropores (Figure 7B3,B4).

### 3.2. Induction Heating-Assisted Resistance Spot Welding

**Figure 8:**
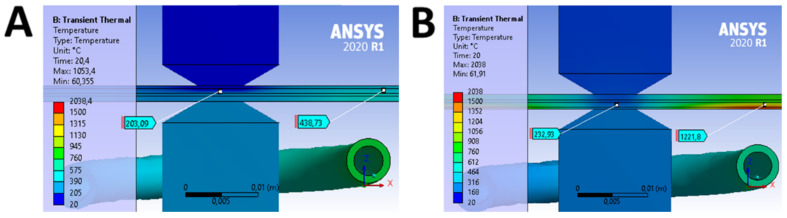
The results of the comparative analysis for two configurations of sheet arrangement: (**A**) solid material of the DP600 was on the side of the inductor and (**B**) MPC material was located on the side of the inductor.

The FEM investigation of the induction heating process was aimed at choosing the proper configuration of the inductor (i.e., inductor placed on the side of solid sheet or MPC side). The analysis showed that the solid material is a kind of thermal buffer that slows down the heating of the MPC. Moreover, it homogenizes the temperature distribution and protects the material from overheating and the polymer from burning. Both cases were analyzed with the same parameters and boundary conditions. Only the positions of the materials were changed so that the solid material of the DP600 was on the side of the inductor (Figure 8A) or so that the MPC material was located on the side of the inductor (Figure 8B).

The results of the obtained analyses clearly show that the location of the MPC material on the side of the inductor caused its very rapid heating, especially the thin (0.3 mm) steel sheet cover. It reached about 1500 °C after 20 s of heating, while the polymer reached the plastic state (232 °C). This temperature was sufficient to allow the free formation flow of the polymer and, most of all, its removal from the welding zone. It should be noted that in this case, the polymer reached almost 1221 °C at the edges of the sheets to be joined. This led to the charring, burning, disintegration, and overall inevitable degradation of the MPC material. Of course, the power used for heating can be reduced, but this prolongs the heating time and the process becomes irrational to other joining methods.

A more advantageous solution is to apply the heating source on the side of the solid material. As the FEM comparative analysis has shown, in this case, the steel cover sheet of MPC was not being overheated and reached a temperature of about 650 °C after 20 s of heating, while the polymer in the welding zone reached a temperature of 203 °C, which was sufficient for further processing. There was also no excessive overheating of the polymer at the edge of the sheets as it reached a temperature of about 439 °C there. For a short heating time, it did not lead to the degradation of the polymer and MPC material as a whole, which was confirmed during metallographic analysis.

Nevertheless, experimental studies have shown that this method did not achieve satisfactory results due to the fact that a large area of the joined elements heats up, causing its delamination and deformation. In the presented configuration, it is not possible to reduce the heating area due to the geometry and the position of the inductor. The implementation potential of this method is similar to the current shunt method.

**Figure 9:**
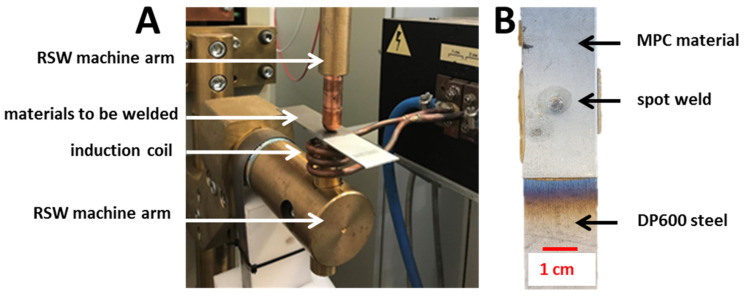
The presentation of: (**A**) sample during induction heating-assisted RSW and (**B**) typical test sample, as welded.

The results of the obtained analyses were compared with the results of the experiments and positively verified. The conducted experimental research allowed us to verify the FEM results. The joining process was properly carried out only for the joint configuration with the solid sheet located on the side of the inductor (Figure 9A). This allowed for the homogenization of the temperature distribution, limiting the risk of delamination and damage to the coating layers of the MPC material. Nevertheless, part of the mass of the polymer core flowed out of the sample (Figure 9B). This means that the greater sample area was heated more than really needed for the further RSW stage. However, this problem will be less significant for larger sheets to be joined, because of the larger distance between the spot weld and the material edge.

**Figure 10:**
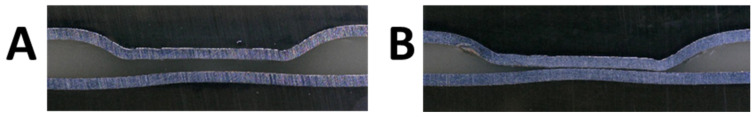
Selection of the induction preheating parameters: (**A**) too short time 20 s and (**B**) correct heating time 30 s.

The welding process was carried out in two stages. In the first stage, the material was preheated by using of the induction method. The parameters for this stage were selected in such a way as to not damage the welded materials (e.g., delamination, polymer burning, or deformation), see Figure 10. The process was carried out at the inductor current *Iz* = 468 A and heating time *t* = 30 s (Figure 10B).

After this stage, the welding machine electrodes were immediately pressed with a force of *F* = 2 kN, and the welding current was turned on. The one pulse welding program with welding current *I* = 5.5 kA during welding time *tz* = 150 ms was used.

**Figure 11:**
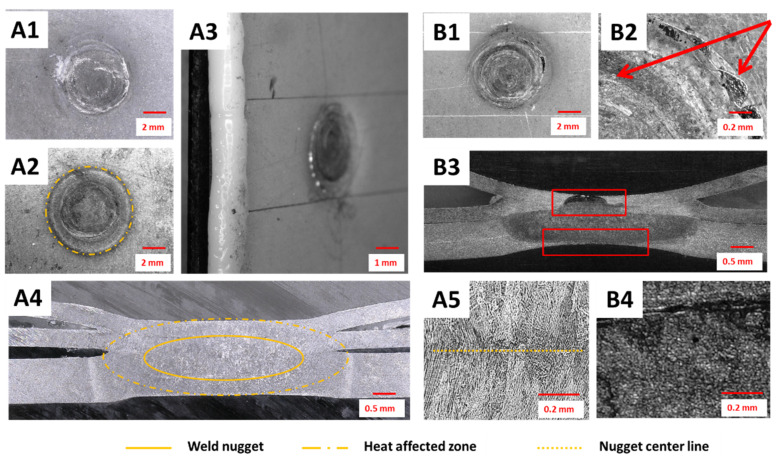
Examples of spot welds manufactured by induction heating-assisted RSW: properly (**A1**) top view, (**A2**) bottom view, (**A3**) side view, (**A4**) cross-section at low magnification, and (**A5**) weld nugget at high magnification and non-properly formulated: (**B1**) top view, (**B2**) top view magnified, (**B3**) cross-section at low magnification, and (**B4**) weld nugget at high magnification.

As a result of the conducted experiments, a group of welded joints with satisfactory properties was made. The average diameter of the joint was 4.7 mm. The general overview of samples properly produced by induction heating-assisted RSW is presented in Figure 11A1–A5. It was possible to obtain a good quality spot weld, with relatively low indentation depth from both sides and without surface breaking cracks. However, a quite extensive heat affected zone was visible (Figure 11A2) as was the polymer core outflow (Figure 11A2). In such a case, the well-formulated weld nugget comprising all metallic layers was produced (Figure 11A4). Due to high heat input during the initial stage of the polymer layer removal, the nugget was built of much coarser grains than in the other two studied concepts (Figure 11A5). On the other hand, usually once the preheating parameters were overestimated, the quality of such spot welds was dramatically decreased, as shown in Figure 11B1–B4. The external steel cover sheet was deformed or even braked. Furthermore, in critical cases, the joint geometry was completely disturbed, resulting from the slip of the MPC cover sheets on the melted polymer core.

The obtained results allow us to state that the method has implementation potential, however, it requires more extensive research on the preheating process. A preliminary preheating stage should be precisely adjusted every time, so that the polymer does not flow out of the MPC material and with no deformation. However, it can be assumed that this concept will still find an application if other preheating methods are not applicable for materials that cannot be heated by ultrasound.

### 3.3. Ultrasonic-Assisted Resistance Spot Welding

The main purpose of the FEM analysis was to compare each of the two methods of ultrasonic heating with the use of transverse and longitudinal vibrations. The analysis of the results was carried out in terms of selecting the heating time and temperature distribution, in the context of achieving the required plasticization temperature of the polymer core. In both FEM models, the direction of vibration was only one parameter to not introduce any additional variables and to ensure constant conditions of comparative analysis.

**Figure 12:**
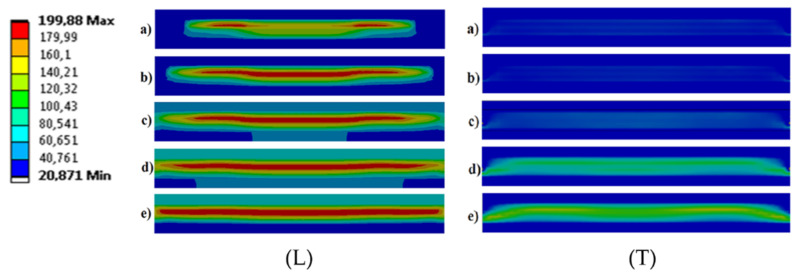
The results of the temperature distribution during ultrasonic heating of the polymer core: (**L**) longitudinal vibration, (**T**) transverse vibration; (a) *ta* = 10 ms, (b) *tb* = 25 ms, (c) *tc* = 50 ms, (d) *td* = 125 ms, (e) *te* = 250 ms.

**Figure 13:**
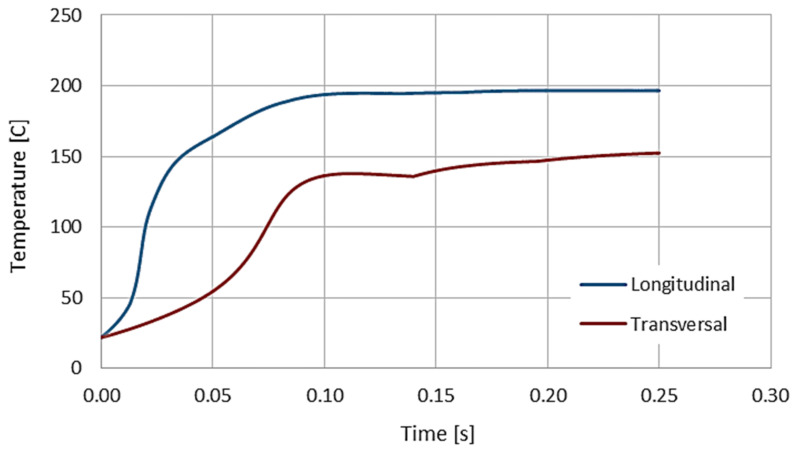
Comparison of the time needed for reaching and the values of the maximum temperatures for the longitudinal and transverse type of vibrations.

The results of the FEM analysis of the ultrasonic heating process showed that the most effective heating method was the UT process with longitudinal vibrations. The temperature distribution in this case was more concentrated in the welding area than in the case of transverse vibrations (see Figure 12). Moreover, this type of vibration ensures the shortest heating time (see Figure 13) without unnecessary heating of the steel cover layers of the MPC.

The polymer core of the Litecor MPC material is mainly composed of a mixture of polyethylene, polyamide [3]. During the preforming of the polymer core of the MPC material, attention should be paid to the appropriate temperature characteristics for polymers. These are mainly the polymer glass transition temperature, *Tg*, and the plasticization temperature, *Tp* (Table 4). The performed numerical analyses showed that by the proper implementation of the ultrasonic heating method, it is possible to obtain temperatures above *Tp* (for a mixture of PA and PE), and thus to form and effectively remove the polymer layer from the welding area.

The heating speed is an essential feature of the discussed longitudinal ultrasonic method. Compared to the other two heating methods (induction and shunt), in this case, thermal energy is generated inside the polymer layer. The problem of the low thermal conductivity of the polymer is not so significant (i.e., the process does not have to be extended to ensure the heat flow in the polymer and its uniform heat distribution). This provides significantly more favorable conditions for the formation (removal) of the polymer layer in the welding area, which is essential from the technological point of view.

**Figure 14:**
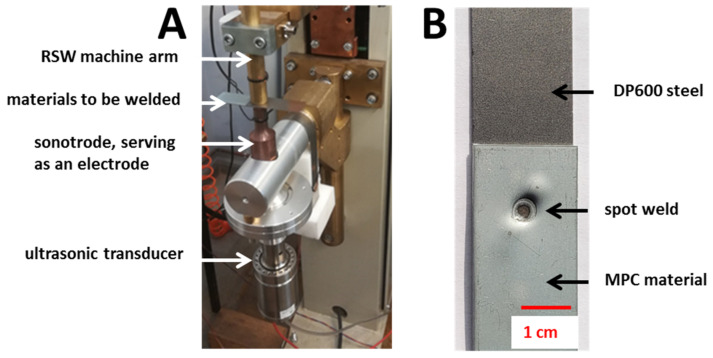
The illustration of: (**A**) UT + RSW process and (**B**) the example of welded joints of Litecor with DP600.

Then, the experimental investigations of the UT-RSW hybrid welding process were carried out (Figure 14). First, the ultrasonic heating parameters were selected to obtain the current contact between the steel shells and the MPC material and to allow the welding current to flow through the area of the joint. If this stage is not performed correctly, it may cause two different effects. In the first case, there is simply no welding current and the joint will be not formulated. The process can then be repeated to produce the correct joint. In the second case, when some welded joints have already been made or there is even a small current contact outside the welding area, the shunt effect may occur. This is a highly unfavorable and dangerous situation for the joined elements. Here, the entire welding current flows through the MPC coating layer. This can lead to overheating, delamination, deformation, and even complete loss of material continuity and expansion. Moreover, the protective layer covering the MPC (e.g., zinc) can also be damaged. For this reason, it is essential to effectively monitor the polymer removal process and the moment of electrical contact. This can be done by classical measurements of dynamic resistance carried out during the ultrasonic heating process. The use of small pulses of the welding current, so as not to damage the welded materials or cause their significant heating, may allow for effective monitoring of this process. It is also possible to provide feedback to the ultrasonic generator to control when the preheating process is completed and the resistance welding process starts. During the selection of the parameters of ultrasonic preheating, it is necessary to shorten the duration of this process as much as possible to decrease the risk of joint damage or delamination of the polymer MPC core.

The UT + RSW joining processes were carried out for three heating times of 150 ms, 250 ms, 1 s, and for five different levels of voltage amplitudes exciting the ultrasonic transducer (i.e., 10%, 20%, 30%, 40%, and 50%), where the maximum voltage amplitude for the transducer used was 1800 V. Two configurations were considered (i.e., the use of an ultrasonic transducer from the solid material side and the other from the MPC material side).

Table 5 shows that the common parameters for both configurations will be the voltage *U* = 720 V (40%) and the vibration time *T* = 250 ms. The resistance welding process was carried out using a two-pulse welding program. The first of the pulses was aimed at removing the contamination layer (i.e., zinc coatings) from the contact surface of the materials to be joined. Moreover, it also allowed us to measure the dynamic resistance value to make sure that the resistance welding process can be safely and properly carried out. The second impulse was used to create a correct weld, covering all steel sheets to be joined. Welding parameters were selected to obtain the maximum diameter of the welding nuggets with the shortest welding time, without causing welding imperfections in the form of expulsions, material surface or coatings, burnout, or joint deformation (Table 6). The average diameter of the joints was 5.3 mm.

**Figure 15:**
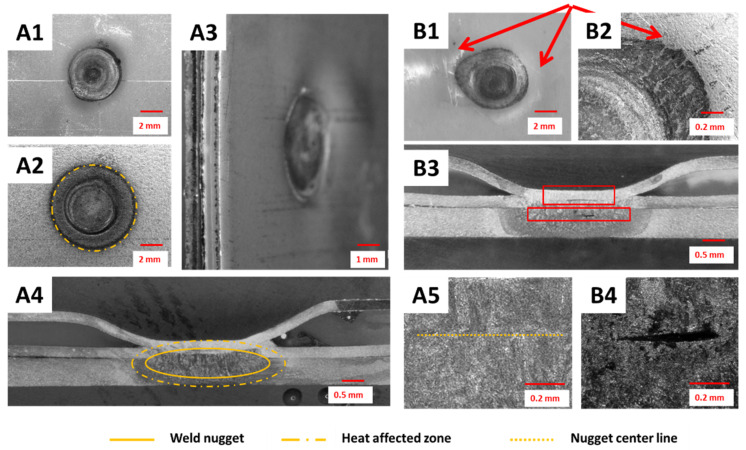
Examples of spot welds fabricated by ultrasonic-assisted RSW: properly (**A1**) top view, (**A2**) bottom view, (**A3**) side view, (**A4**) cross-section at low magnification, and (**A5**) weld nugget at high magnification and non-properly formulated (**B1**) top view, (**B2**) top view magnified, (**B3**) cross-section at low magnification, and (**B4**) weld nugget at high magnification.

The macro- and microstructural observations of the UT + RSW joints showed that it is easily possible to formulate a proper spot weld (Figure 15A1–A5). In such cases, the weld nugget was properly formulated and covered all three steel sheets (MPC cover sheets and the DP600 steel sheet). The heat affected zone was very narrow, much smaller than in the two other concepts; similarly, the grains were of small size in the central part of the weld nugget (Figure 15A4,A5). The polymer core was well removed and heated locally, so the core layer surrounding the spot weld was free of pores and discontinuities. However, this also caused some nonregular deformation of the outer layer of the MPC cover sheet (Figure 15A3,B1,B2). Defects in spot welds, in this specific method, occurred rarely, however, the most common were the lack of fusion between all metal layers or small shrinkage cavities observed in the center of the weld nugget (Figure 15B3,B4).

In order to summarize the experimental work performed, the typical parameters of hybrid, RSW welding-based processes are presented in Table 7. In case of incorrect parameters (too “low” or too “high”) at any stage of the process, many significant non-conformities appeared in the joint’s structure. However, it should be clearly stated that in such hybrid processes, as presented in this work, there are many different factors like the condition of the material surface, geometry of the electrodes, the stability of the welder control system, the conditions of heat dissipation from the welding area, etc., which significantly affects the whole joining process and the final weld. Therefore, the values given in Table 7 should be considered illustrative.

## 4. Conclusions

In this work, the possibility of joining the metal–plastic composite (i.e., Litecor) to DP600 steel was investigated. Three various resistance spot welding-based processes were developed and tested. The polymer core was first heated by means of an (i) electric current shunt circuit; (ii) induction heating; or (iii) high intensity ultrasonic waves, and then removed from the welding zone. Afterward, the proper RSW joining process could occur. Based on this research work and the MPC joining process development, the following conclusions may be formulated:

- Shunt current-assisted RSW: an appropriate design of the current shunting element and the accurate selection of the initial process parameters is needed, as there is a high risk to overheat the MPC material. To lower the risk of degradation of the MPC material, the multi-pulse process may be applied. The most common joint defects observed here were splash or cracking of the outer metal sheet in MPC, followed by an extensive polymer expulsion, when the process parameters were not properly set. Due to the need to mount the shunt current element, it is rather difficult to use this concept in an automated mode;
- Induction heating-assisted RSW: this allows for the most effective heating of the materials in the welding zone, out of all testing methods. However, the proper placement of materials on the inductor is needed to obtain homogeneous temperature distribution and to protect the MPC material from rapid and extensive overheating. Nevertheless, a big part of the polymer core flowed out as a relatively big area of material was heated at once. As a result, a quite extensive heat affected zone is visible in the formulated joint and if the induction heating process is too long, then the overall quality of the joint dramatically decreases; and
- Ultrasonic-assisted RSW: this is the most complex concept. It requires proper UT knowledge, as the type of UT vibration (here: transverse and longitudinal) strongly influences the heating of materials in the joining zone. The UT stage is very short, so it does not cause the extension of the entire joining cycle process, which is essential in terms of further development and application. The ultrasonic-assisted RSW allows for joining the materials very locally; on the other hand, this also causes some nonregular deformation of the outer layer of the MPC cover sheet just next to the joint.

## Figures and Tables

**Table 1:** Chemical composition (max. values) of HX220YD steel (in wt.%; acc. to EN 10346) and of DP600 steel (in % by weight; acc. to EN 10338) [25,27,28].

Material	C	Si	Mn	P	S	Al	Nb	Ti	Cu *	Cr + Mo	V
HX220YD	0.01	0.2	0.9	0.08	0.025	0.1	0.09	0.12	0.2	-	-
DP600	0.17	0.8	2.2	0.08	0.015	2	Nb + Ti	0.15	-	1	0.2

* According to VDA 239-100.

**Table 2:** Mechanical properties of HX220YD steel (acc. to EN 10346) and of DP600 steel (acc. to EN 10338) [25,28].

Property	Yield Strength, R_0.2_ MPa	Ultimate Tensile Strength. R_m_ MPa	Total Elongation, A_80_ %
HX220YD	220–280	340–420	32
DP600	340–420	600	20

**Table 3:** Selected boundary conditions and physical properties.

Parameter/Material	Value	Unit
**General for all materials**		
Convective heat transfer coefficient	20	W/(m^2^ × K)
Contact heat transfer coefficient	1000	W/(m^2^ × K)
Emission coefficient	0.6	-
**Steel**		
Thermal conductivity	60.5	W/(m × K)
Specific heat	434	J/(kg × K)
**Polymer**		
Thermal conductivity	0.28	W/(m × K)
Specific heat	2300	J/(kg ×K)

**Table 4:** Characteristic temperatures for the selected polymers [31].

Material Type	Polyethylene	Polyamide (PA6)	Polypropylene
Glass trans. temp. *Tg* [°C]	−125	50	−10
Plasticization temp. *Tp* [°C]	110	225	165

**Table 5:** The selection of the ultrasonic heating parameters.

Configuration	MPC from the Sonotrode Side			DP600 from the Sonotrode Side		
*U* [V]/*T* [s]	0.150	0.250	1	0.150	0.250	1
10%	0	0	0	0	0	0
20%	0	0	x	0	0	x
30%	0	x	x	0	0	x
40%	x	x	x	0	x	x
50%	x	x	x	0	x	x

x—current contact, 0—no contact, background color—best parameters tested.

**Table 6:** The selected parameters of the welding process.

Current Impulse Stage	Electrode Force F (N)	Welding Current (kA)	Welding Current Flow Time (s)
First impulse	1000	3.1	0.15
Second impulse	1000	5.5	0.15

**Table 7:** The summary of the typical parameters of hybrid RSW processes.

Process Type	Stage 1—Polymer Removal	Stage II—RSW Process
**Shunt Current-Assisted RSW**	**Too low parameters:** *Is* < 3.0 kA (current of the single pulse), *ts* < 130 ms (time of single pulse), *Ns* < 3 (number of current pulses), 🢂 No electrical contact of welded sheets, RSW process impossible **Correct parameters:** *Is* < 3.5 kA, *ts* < 150 ms, *Ns* < 5, 🢂 Good electrical contact of welded sheets, RSW process possible **Too high parameters:** *Is* > 4.0 kA, *ts* > 170 ms, *Ns* > 10, 🢂 Overburn of the MPC covering sheet, leak of the polymer outside, expulsion, MPC delamination, high degree of material deformation	**Too low parameters:** *Iz* < 5.5 kA, *tz* < 130 ms, *Fz* < 0.8 kN, 🢂 Too small welding nugget, low strength, **Correct parameters:** *Iz* = 6 kA, *tz* = 150 ms, *Fz* = 1 kN, 🢂 The welding nugget > 3.5 mm, strength > 2.5 kN **Too high parameters:** Iz > 6.5 kA, tz > 180 ms, Fz > 1.5 kN, 🢂 Too deep indentation, expulsion, irregular welding nugget, over burned weld surface, low fating strength
**Induction Heating-assisted RSW**	**Too low parameters:** I < 420 A, t < 20 s, 🢂 No electrical contact of welded sheets, RSW process impossible **Correct parameters:** I = 468 A, t = 30 s, 🢂 Good electrical contact of welded sheets, RSW process possible **Too high parameters:** I > 500 A, t > 35 s, 🢂 Over burn of the polymer core, leak of the polymer outside, expulsion, MPC delamination, high degree of material deformation	
**Ultrasonic-Assisted RSW**	**Too low parameters:** Au < 540 V (the amplitude of transducer supply), tu < 150 ms (the time of ultrasonic pulse), 🢂 No electrical contact of welded sheets, RSW process impossible **Correct parameters:** Au = 720 V, tu = 250 ms, 🢂 Good electrical contact of welded sheets, RSW process possible **Too high parameters:** Au > 900 V, tu > 1 s, 🢂 Over burn of the polymer core, leak of the polymer outside, expulsion, MPC delamination, high degree of material deformation	

## Data Availability

Data sharing is not applicable to this article.
